# Thyrotoxicosis antidote assay along with concurrent medication; chromatographic and environmental issues

**DOI:** 10.1186/s13065-025-01487-1

**Published:** 2025-05-12

**Authors:** Raghda A. Emam, Aml A. Emam, Rehab M. Abdelfatah, Basma H. Anwar

**Affiliations:** Pharmaceutical Analytical Chemistry Department, Faculty of Pharmacy, Beni-Suef University, Alshaheed Shehata Ahmad Hegazy St., Beni-Suef, 62514 Egypt

**Keywords:** Greenness assessment, Hydrocortisone acetate, Methimazole, Thin layer chromatography, Propranolol, Spiked human plasma

## Abstract

A thionamide medication, Methimazole (MTZ), is a crucial antidote of thyroid hormone in cases of toxic nodular goiter or thyrotoxicosis by decreasing thyroid hormone synthesis. Propranolol (PRP), a beta blocker, is commonly co-administered with MTZ for controlling tachycardia associated with hyperthyroidism. Quantitative determination and tracing of MTZ in plasma in the presence of its co-administered medication, like PRP, is of paramount importance due to the reported toxic manifestations related to MTZ long-term administration that include agranulocytosis, hepatitis, arthritis, and skin rashes. An environmentally benign FDA-validated TLC densitometric approach was established for the first time for simultaneous and quantitative analysis of MTZ and PRP in pure form and spiked human plasma. The work is considered a mimetic study for their co-administration. Successful resolution between them was achieved on Merck^®^ silica gel plates using a mixture of ethyl acetate: acetone: 33% NH_3_ solution in a ratio of (9: 1: 0.05, by volume) as a developing phase and UV scanning at 254 nm. Adding hydrocortisone acetate (HCA) as an internal standard eliminated the matrix effect variation. Reasonable resolution of the developed peaks was attained, with R_f_ values of 0.07, 0.19, 0.67, and 0.81 for plasma, PRP, MTZ, and HCA, respectively. Four greenness and viability rating approaches were applied to check and measure the greenness aspects of the suggested method toward the eco-system, and the outcomes were convenient and satisfactory. Also, the verification domains were tested using US-FDA bio-analytical specifications where reliable and acceptable outcomes were obtained with satisfied % recoveries for the quality control samples ranging from (100.39–104.44%) and (96.01–100.72%) for MTZ and PRP, respectively, with low RSD values indicating good accuracy and precision of the proposed method.

## Introduction

**Fig. 1:**
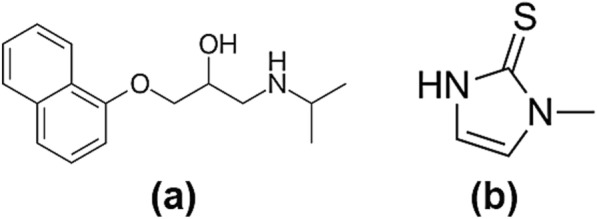
The chemical structures of **a** propranolol and **b** methimazole

Methimazole (MTZ), Fig. 1, is a thionamide medication used for managing hyperthyroidism, which is a medical condition characterized by over-production of thyroid hormone (thyrotoxicosis), leading to health complications [1]. These complexities include atrial fibrillation (with its consequent complications such as stroke and heart failure), in addition to osteoporosis, hard conceiving, miscarriage, menstrual disturbances, muscle weakness, and Graves'disease, which is associated with light sensitivity and double blurred vision [2]. MTZ works by suppressing the thyro-peroxidase enzyme, which is responsible for thyroid hormones formation, leading to the inhibition of thyroid hormones synthesis, including thyroxine (T_4_) and triiodothyronine (T_3_), which results in restoring normal thyroid function [1].

Propranolol (PRP), Fig. 1, is a β-adrenoceptor antagonist [3]. It is used as a treatment for controlling hypertension, myocardial infarction, arrhythmia, and angina. It is also used to manage hyperthyroidism, anxiety disorders, and hyperthyroid tremor in case of sympathetic overactivity cases [4].

PRP and MTZ are co-administered for hyperthyroidism treatment, as MTZ can optimize thyroid hormone production and enhance hepatic function. At the same time, PRP can adjust the heart rate and improve the health quality of the patient, with the lowest side effects [5]. Unfortunately, long-term administration of MTZ was found to be related to agranulocytosis, toxic hepatitis, arthritis, and skin rashes symptoms [1].

In the literature, MTZ was analyzed and determined using spectroscopic [6, 7], TLC [8], HPLC [9–12], GC [13], and voltammetric [14, 15] methods in different samples and conditions. In addition, PRP was analyzed and determined using spectroscopic [16–19], TLC [20, 21], HPLC [22–26], and voltammetric [27, 28] methods in different samples and conditions.

MTZ and PRP had not been determined simultaneously in any published work. Such toxic manifestations created an increasing demand for developing an accurate and sensitive assay method for MTZ quantification along with PRP, which improves its performance, especially in case of a lack of any published analytical approach for their simultaneous determination upon review of the literature. Therefore, the purposes of this article are to develop an environmentally benign TLC assay method for quantifying and separating PRP and MTZ in pure form and spiked human plasma, to verify the suggested method in compliance with US-FDA specifications, and to assess the proposed method's environmental impact and viability by applying various evaluating tools.

In addition, four greenness and viability rating approaches were applied to check and assess the greenness aspects of the suggested method toward the eco-system, namely: eco-scale assessment (ESA) [29], green analytical procedure index (GAPI) [30], analytical greenness metric approach (AGREE) [31], and practicability metric approach (BAGI) [32]. Also, the whiteness of the method was appraised using the red–green–blue (RGB) Excel sheet [33]. The outcomes were convenient and satisfactory, showing that the proposed TLC method is acceptably green and eco-friendly to the environment and human lives.

## Experimental

### Instruments

#### For TLC—densitometric approach

- TLC (Linomat V) applicator was used to apply samples with a 100 µL Camag (Muttenz, Switzerland) syringe.
- The detection of the resulting peaks was achieved with the assessment of a TLC densitometer scanner (Camag, Switzerland) by adjusting the following parameters (the mode of scanning: absorbance, the slit measures: 6 × 0.45 mm, the scanning speed: 20 mm/s, and the radiation source: a deuterium lamp.
- The software used was WinCATS (Camag, version 3.15), which was connected to a TLC scanner to record and manipulate the resulting data.
- Merck^®^ Silica gel-coated aluminium plates 60 F254 (25 × 10^–2^ mm thickness) served as stationary phase, and the plate dimensions were (20 × 20 cm).

#### For plasma sample preparation

- Digital balance (Sartorius, German) was used to withdraw the accurate weight of the sample powders.
- Different sample volumes were withdrawn using Rongtai micropipette devices with various volumes of 0.1–100 µL (Shanghai, China).
- An ultra-sonicator (Sonix TV SS-serious) was used to sonicate the samples (New York, USA).
- A Low-speed 80–2 C electric centrifuge (4000 rpm) with 12 × 20 mL capacity and 110 v/220v power supply (Zjmzym, China) was used to centrifuge the prepared samples.
- Syringe filters with 45 × 10^–2^ µm pore size (Npore Ghaziabad, India) were utilized to filter the samples.
- A vortex mixer (Hwashin, Seoul, Korea) was employed to mix the samples.

### Materials

#### Pure standards

- PRP and hydrocortisone acetate (HCA) with certified purities of (99.21 and 99.63%, respectively) were provided by Al-Kahira Company for Pharmaceuticals (Giza, Egypt).
- MTZ, with a certified purity of 99.53, was bought from Sigma Aldrich (Cairo, Egypt)

#### Chemicals and reagents

- Methanol (Fischer, UK) of HPLC grade was utilized.
- Ethyl acetate, acetone, and 33% NH_3_ solution of analytical grade were bought from AL-Nasr Pharmaceutical and Chemical Company (Cairo, Egypt).
- No prior purification was employed for the chemicals and solvents utilized.

### Standard solutions and blank plasma sample

- Stock solutions of 1000 µg/mL of PRP, MTZ, and HCA were made separately in three 10-mL volumetric flasks, using methanol as solvent.
- Further working solutions of 100 µg/mL and 10 µg/mL of PRP and MTZ were made separately from the previously made stock solutions into two different 10-mL volumetric flasks using the same solvent.
- For the blank plasma samples, after transferring 1 mL of drug-free plasma into a 10-mL measuring flask, methanol was added to adjust the volume to 10 mL. Also, plasma proteins were precipitated before collecting the clear supernatant, and the sample was centrifuged.

## Procedures

### Chromatographic parameters

Samples were applied to the TLC plates using a Camag Linomat IV applicator in bands (three mm wide) spaced six mm from each other and ten mm apart from the bottom border. The mobile system was made up of ethyl acetate: acetone: 33% NH_3_ solution (9: 1: 0.05, by volume), which was put into a jar made of glass and left for saturation for 10 min. After that, the plates were put into the jar, tightly sealed, and left until the mobile system reached the plate's front line, one centimeter in front of the plate's end. In the end, UV scanning was employed on the dry plates to detect the resulting peaks spectrophotometrically at 254 nm. The samples were applied with a volume of 10 µL.

### Calibration graphs construction and preparation of quality control samples

#### For pure samples

Using their respective stock solutions, two groups of 10-mL volumetric flasks were filled with varying concentrations between (0.04–1.0) µg/band for PRP and (0.1–1.0) µg/band for MTZ. Each flask received 0.1 mL of the internal standard (HCA) solution (1 mg/mL) before the volume was marked with methanol. Subsequently, 10 µL injections of each sample were applied in triplicate, and the procedures listed under"chromatographic conditions"were employed. Ultimately, calibration curves were created by plotting the integrated peak area ratios (peak area of the analyte/peak area of internal standard) against the corresponding concentration in µg/band.

#### For spiked human plasma samples

In the limits of (0.06–1.0) and (0.1–1.2) µg/band of PRP and MTZ, respectively, various concentrations were created utilizing their corresponding stock and working solutions into two sets of 10-mL measuring flasks. Each flask received 0.5 mL of internal standard (HCA) stock solution and 1 mL of drug-free plasma, and then the volume was completed with methanol. After 5 min sonication of the samples, 5 min centrifugation was performed to exclude the precipitated plasma proteins, and the clear supernatant was applied in 10 µL volume on the TLC plates as triplicates following the previously outlined"chromatographic conditions."At the end, the calibration curves were computed by graphing the integrated peak area ratios (peak area of the analyte/peak area of internal standard) of the spiked human plasma samples and the corresponding concentration in µg/band. In addition, as FDA specifications stated, four quality control samples should be prepared and analyzed employing a similar procedure for both drugs [34].

## Results and discussion

Thin-layer Chromatography is a reliable lab method for separating and examining different components in a mixture. Its various benefits include taking little in the way of tools and reagents, making it reasonably simple and inexpensive. TLC provides rapid results, allowing the speedy identification and separation of mixture components. It is also suitable for rare or precious samples because it only requires small sample volumes. Additionally, TLC can easily be applied to both qualitative and quantitative evaluations. Its adaptability in disentangling various medications and constituents from intricate matrices amplifies its usefulness in experimental settings [35–38]. Therefore, this study offers a newly verified TLC method for simultaneous analysis and quantification of MTZ, which is reported to be toxic upon long-term administration [1], in addition to PRP, which synergizes its action in their binary mixture and spiked human plasma. It has the benefits of being environmentally green, quick, easy, inexpensive, and requiring little amounts of samples and reagents.

### Method optimization

#### The selection of the developing phase composition and ratio

**Fig. 2:**
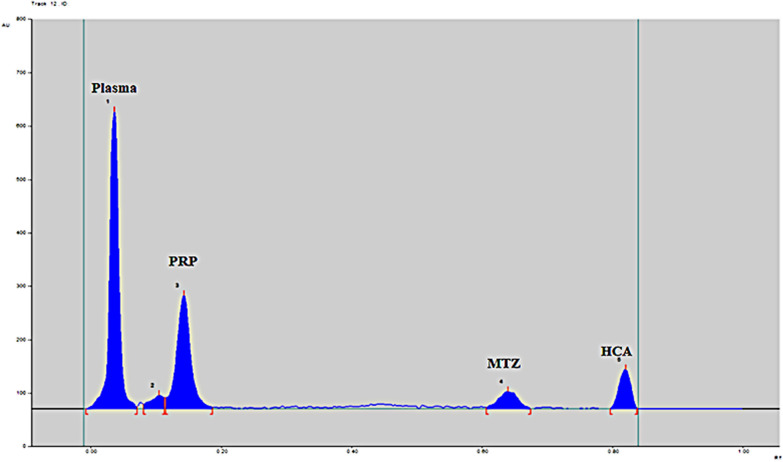
2D chromatogram of non-fully optimized chromatographic conditions showing insufficient resolution between tailed PRP peak and that of plasma and high R_f_ value (0.83) of HCA peak, close to the solvent front, using a developing system of ethyl acetate: acetone (9: 1, by volume) at 254 nm

Different developing solvents were tested to separate MTZ and PRP from plasma for optimum selectivity and resolution. Different developing solvents with different ratios were tested: methanol (MTH): acetone (AC), ethyl acetate (EA): AC and EA: MTH in the ratios of 6: 4, 4: 6, 7: 3, 3: 7, 9: 1, 1: 9. A developing system of MTH: AC (6: 4, by volume), resulted in separated PRP and MTZ peaks with tailed PRP one. Another trial was carried out by replacing methanol with EA; as greener than it [39], a developing system of EA: AC (6:4, by volume) was used that improved PRP peak symmetry, but the MTZ band appeared near the solvent front. Increasing EA proportion to be EA: AC (9: 1, by volume) attained the best peaks'symmetry, with MTZ peak away from the solvent front, but PRP'one appeared near the baseline adjacent to the plasma band. At this step, different drugs (hydrocortisone, HCA, glibenclamide, and etoricoxib) were tested to be used as internal standards, applying the last developing system. Etoricoxib and hydrocortisone bands appeared at Rf values close to that of MTZ. At the same time, GLB one was near baseline, and HCA appeared near the front line but separated from the MTZ peak, so it was chosen as an internal standard, as shown in Fig. 2. Finally, ammonia solution (33%) was added to the system (EA: AC, NH_3_, 9: 1: 0.05, by volume), led to good separation of PRP peak away from that of plasma and the baseline and achieved reasonable separation between MTZ and HCA away from front line while attaining optimum peaks symmetry. Accordingly, the final developing system was EA: AC: 33% NH_3_ solution in a ratio of (9: 1: 0.05, by volume).

#### The selection of the detection wavelength

Different scanning trials used other wavelengths, including 230, 254, and 260 nm. The optimum detection wavelength was 254 nm, with the best sensitivity and lowest noise.

#### The selection of the internal standard

The chromatographic separation and performance were optimum after trying different drugs as internal standards, such as hydrocortisone, HCA, glibenclamide, and etoricoxib. They attained the best results when using HCA as an internal standard. It was the best one for compensating for changes in extraction efficiency, and its peak shape was symmetric and separated with good resolution from the peaks of other components under the same conditions.

#### Slit dimensions of scanning light beam

**Fig. 3:**
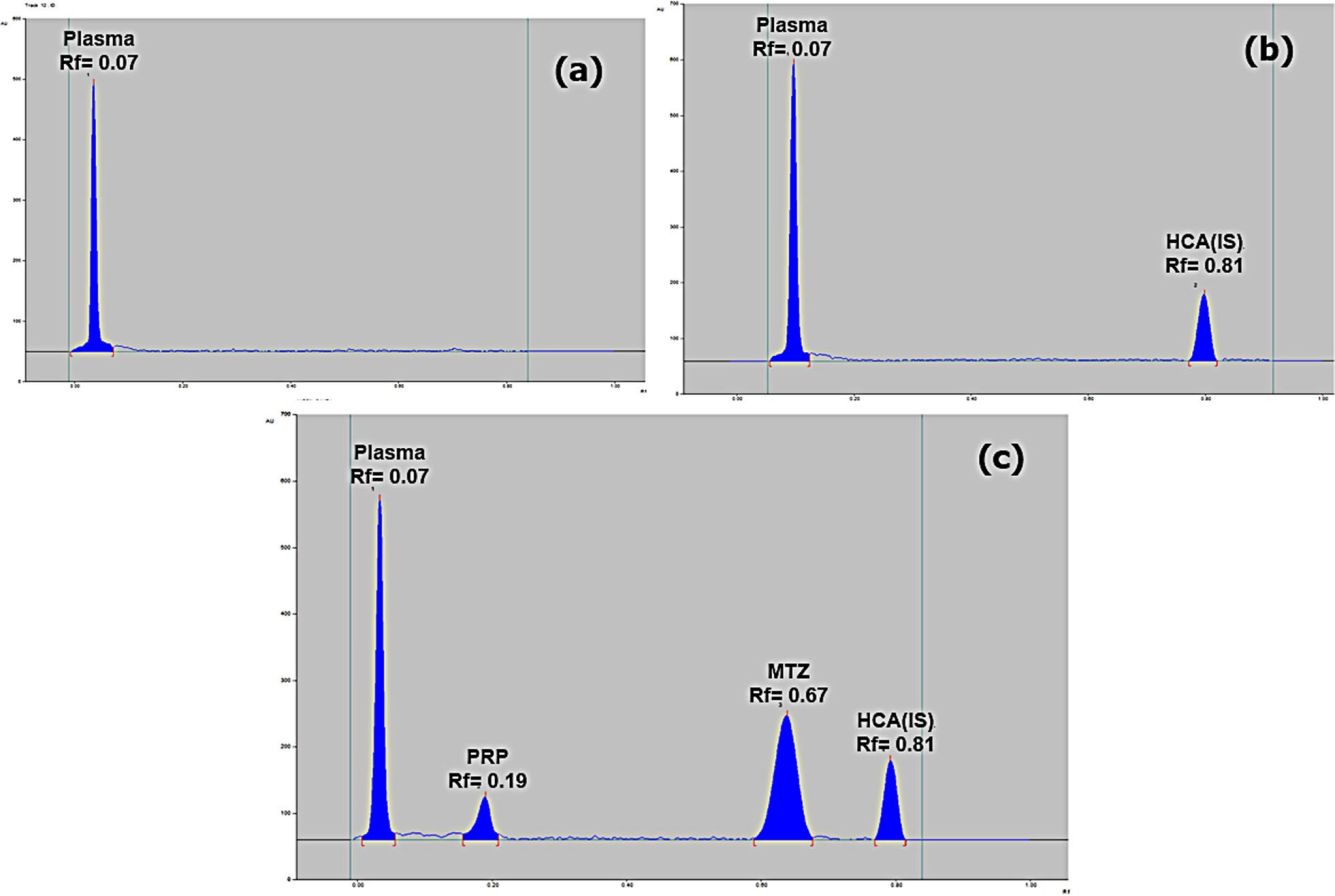
2D chromatogram of **a** blank human plasma, **b** plasma and 0.5 µg/band of the internal standard hydrocortisone acetate, **c** spiked human plasma with 0.8 µg/band of propranolol, 1.0 µg/band of methimazole and 0.5 µg/band of the internal standard hydrocortisone acetate, using ethyl acetate: acetone: 33% NH_3_ solution (9: 1: 0.05, by volume) as a developing system at 254 nm

The band dimensions were tested and enhanced by optimizing the slit dimensions of the scanning light beam. The interference of the adjacent band should be eliminated. Different trials of slit dimensions were carried out. The slit dimension of (6 × 0.45) mm was found to be the optimum one with the best sensitivity. Finally, the studied drugs were well separated at R_F_ values of 0.07, 0.19, 0.62, and 0.81 for plasma, PRP, MTZ and HCA, respectively using ethyl acetate: acetone: 33% NH_3_ solution in a ratio of (9: 1: 0.05, by volume) as a developing system, HCA as internal standard, 254 nm as UV-scanning wavelength and slit dimension of (6 × 0.45) mm, as shown in Fig. 3.

### Method development

**Fig. 4:**
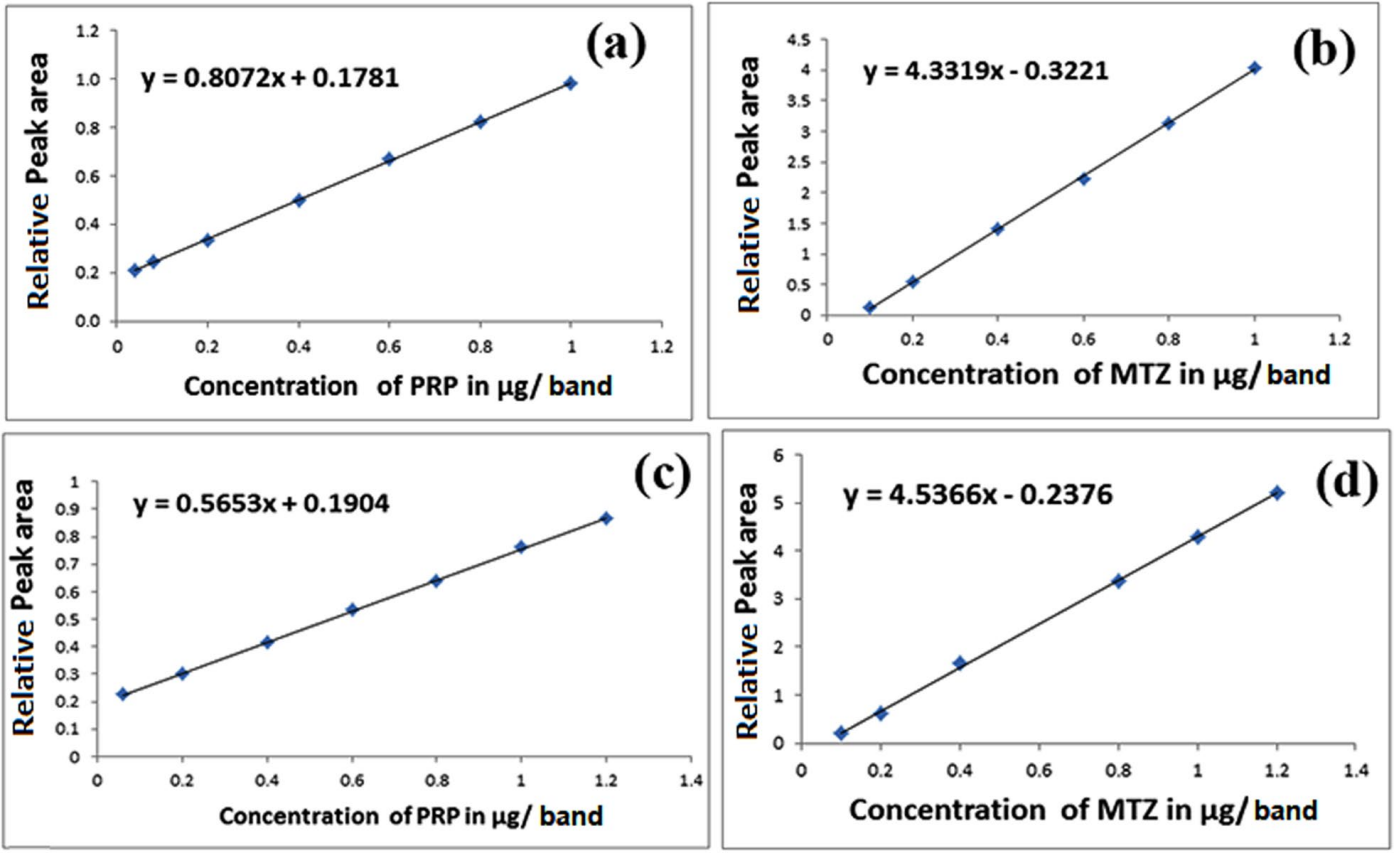
Calibration curves relating the peak area ratios of **a** propranolol in pure form to its concentrations in the range 0.04–1.0 μg/band, **b** methimazole in pure form to its concentrations in the range 0.1–1.0 μg/band, **c** propranolol in spiked human plasma to its concentrations in the range 0.06–1.2 μg/band, **d** methimazole in spiked human plasma to its concentrations in the range 0.1–1.2 μg/band using the proposed TLC method

Plotting the peak area ratio (peak area of the drug/peak area of IS) against the corresponding concentration in µg/band allowed for constructing the calibration curves, which were then created following the procedures outlined in the"chromatographic conditions"section. As illustrated in Fig. 4, linear regressions were seen in the concentration ranges of 0.04–1.0 µg/band for PRP and 0.1–1.0 µg/band for MTZ. Table 1 provides the criteria for the regression equations. As seen in Fig. 3c, the obtained R_f_ values for plasma, PRP, MTZ, and HCA were 0.07, 0.19, 0.62, and 0.81, respectively.

**Table 1:** Assay and method validation parameters for the determination of propranolol and methimazole by the proposed TLC method

Parameters	Pure		Spiked human plasma	
	PRP	MTZ	PRP	MTZ
Calibration range (µg/band)	0.04–1.0	0.1–1.0	0.06 −1.2	0.1–1.2
Slope	0.8072	4.3319	0.5653	4.5366
Intercept	0.1781	− 0.3221	0.1904	− 0.2376
Correlation coefficient	0.9998	0.9998	0.9997	0.9996
Accuracy	99.64	100.26	–	
Robustness parameters (RSD %) ^a^ - 33% NH_3_ solution (0.05 ± 1%) - Acetone (1 ± 1% mL) - Saturation time (10 ± 5 min)	–		1.517 0. 698 1.982	1.479 1.257 1.730
LLOQ (µg/band)			0.06	0.1
ULOQ (µg/band)			1.2	1.2

^a^ the %RSD was calculates for the R_f_ values

### Method validation

The developed bio-analytical TLC method was validated by adhering to FDA [34] as follows:

#### Calibration curves, lower and upper limits of quantification (LLOQ & ULOQ)

For PRP and MTZ, linear calibration curves were produced with concentration ranges of 0.06–1.2 µg/band and 0.1–1.2 µg/band, respectively, after plasma samples spiked with the medications under study. As indicated in Table 1, the LLOQ and ULOQ were detected to be 0.06 and 1.2 µg/band for MTZ and 0.1 and 1.2 µg/band for PRP.

#### Accuracy, precision, and quality control samples (QCs)

LLOQ, LQC, MQC, and HQC were the four QC samples validated to assess the established procedure's precision and accuracy. For PRP, (0.06, 0.2, 0.4, and 1.0) µg/band and (0.1, 0.3, 0.6, and 1.0) µg/band for MTZ were the chosen QC concentrations. The concentrations of the medications under study were calculated using the matching regression equations provided in Table 1, and the results were deemed acceptable, as indicated in Table 2.

**Table 2:** Intra and inter-assay precision and accuracy of LLOQ, LQC, MQC, and HQC of propranolol and methimazole in spiked human plasma samples

Component	Concentration (µg/band)^a^		Intra-day			Inter-day		
			Recovery %	RSD %	Bias %^b^	Recovery %	RSD %	Bias %^b^
PRP	LLOQ	0.06	103.61	2.090	3.61	102.83	2.971	2.83
	LQC	0.2	104.44	3.990	4.44	98.72	4.018	− 1.28
	MQC	0.4	100.39	1.890	0.39	102.54	3.111	2.54
	HQC	1.0	103.30	1.040	3.30	99.28	2.882	− 0.82
MTZ	LLOQ	0.1	96.01	1.798	− 3.99	102.24	3.178	2.24
	LQC	0.3	96.65	2.820	− 3.35	104.85	4.281	4.85
	MQC	0.6	98.35	1.987	− 1.65	102.07	2.753	2.07
	HQC	1.0	100.72	3.059	0.72	97.21	4.071	− 2.79

^a^ Average of 3 experiments

^b^ Bias = [(measured concentration—nominal concentration)/nominal concentration] × 100

#### Specificity and selectivity

By comparing the TLC chromatograms produced from the application of drug-free plasma samples (from six different plasma batches) and spiked plasma samples containing the two investigated medications at their LLOQ and IS, the specificity of the sample was verified. The TLC chromatograms in Fig. 3 demonstrated the new method's selectivity, displaying a good separation between plasma, PRP, MTZ, and the internal standard HCA without interference from the plasma matrix and plasma proteins.

#### The freeze–thaw and benchtop stability

The stability of the drugs under study was assessed using three freeze–thaw cycles and benchtop stabilities, and the results were acceptable and satisfactory. They demonstrated the stability of the drugs under study, as indicated in Table 3.

**Table 3:** Stability results of propranolol and methimazole in spiked human plasma at different conditions using the proposed TLC method

The drug	Recovery %^a^		
	Concentration (µg/band)	Three freeze–thaw cycles^b^	Benchtop stability
PRP	0.2	96.08	97.55
	0.4	104.47	98.46
	1.0	103.78	100.22
Mean ± % RSD		101.44 ± 4.591	98.74 ± 1.375
MTZ	0.3	101.96	102.27
	0.6	104.88	103.721
	1.0	99.94	100.68
Mean ± % RSD		102.26 ± 2.429	102.22 ± 1.487

^a^ Average of 3 determinations

^b^ Freezing was done at −20 ºC

#### The extraction recovery

The extraction recovery for the proposed drugs was calculated and analyzed by matching the areas beneath the peaks of the extracted samples at LQC, MQC, and HQC concentrations versus extracts of blanks spiked with the analyte post-extraction at the same concentration levels [34]. The results in Table 4 confirmed that the current work's extraction method was accurate.

**Table 4:** The extraction recovery results of propranolol, methimazole and hydrocortisone acetate in spiked human plasma

PRP		MTZ		HCA	
Concentration (μg/band)	% Recovery^a^	Concentration (μg/band)	% Recovery^a^	Concentration (μg/band)	% Recovery^a^
0.2	106.87	0.3	93.18	0.4	95.16
0.4	102.41	0.6	95.96		93.95
1.0	99.61	1.0	96.51		92.72
Mean ± % RSD	102.96 ± 3.56	95.22 ± 1.87		93.94 ± 1.300	

^a^ Average of 3 determinations

#### System suitability parameters

The resolution, tailing, and selectivity factors were among the many metrics calculated to assess the system's suitability. The results were validated per the permitted ranges, as shown in Table 5 [40].

**Table 5:** System suitability parameters of the developed TLC method for the determination of propranolol and methimazole

Parameters	Plasma	PRP		MTZ		HCA	Reference value [38]
Retardation factor (R_f_)	0.07	0.19		0.67		0.81	–
Capacity factor (K')	–	4.26		0.49		0.23	0–10
Symmetry factor	–	1.00		1.04		1.00	~ 1
Resolution (Rs)	5.63		10.90		2.52		R > 1.5
Selectivity (α)	3.12		8.69		2.13		α > 1

#### Robustness

By making minor, carefully considered adjustments to several parameters, including the developing system proportions, the scanning wavelength, and the saturation period, the robustness of the devised TLC method was examined, and the findings were recorded in Table 1.

### Greenness assessment of the developed TLC method

#### Eco-scale assessment (ESA)

The analytical eco-scale is one of the authorized metrics for assessing the process's environmental friendliness [29]. Table 6 displays the eco-scale ratings determined for the established TLC. The findings demonstrate that, per the Globally Harmonized System of Classification and Labeling of Chemicals (GHS), the established approach got an eco-scale grade of 80, explaining that the suggested TLC method is acceptably green [29] with minimum impact on the environmental life and human health.

**Table 6:** Eco-scale penalty points of the developed TLC method for simultaneous determination of propranolol and methimazole in spiked human plasma

Eco-scale assessment				
The reagents				
Items	Amount		Hazard^b^	Total penalty points^c^
Acetone	1 (3 ~ < 10 mL)		2 (1 pictograms, danger)	2
Ethyl acetate	2 (27 ~ 10–100 mL)		4 (2 pictograms, danger)	8
NH_3_ solution	1 (0.05 ~ < 10 mL)		6 (3 pictograms, danger)	6
The Instruments				
Energy used		1 (LC < 1.5 kWh per sample)		
Occupational hazard		0		
Waste^a^		3 (1.5 ~ 1–10 mL)		
Total penalty points		Σ 20		
Analytical Eco-scale score		80		

GAPI pictogram	AGREE pictogram	BAGI Pictogram
		
RGB model		


^a^ Waste = the volume of mobile phase/No. of spots per TLC plate

^b^ Hazard penalty points = No. of pictograms × signal. The signal maybe warning = 1 or danger = 2

^c^ The total penalty points = the amount penalty points × hazard penalty points

#### Green analytical procedure index (GAPI)

A reliable resource that offers an extensive environmental assessment of the entire analytical methodology, from the collection of the sample to the final results, is the GAPI tool [30]. Table 6 presents an evaluation and illustration of the five primary GAPI pictogram sections of the proposed TLC technique, composed of four green, nine yellow, and two red segments. The results proved that the proposed method is acceptably green with minimum impact on environmental life and human health.

#### Analytical greenness metric approach (AGREE)

The AGREE tool, an efficient online method for evaluating green characteristics [31], can be downloaded from a defined online link listed in the AGREE research [31]. Table 6 illustrates and displays the colored pictogram and total AGREE score for the developed TLC method, which is equal to 0.73 with a light green shade, which indicates the greenness of the developed TLC method and that it is an eco-friendly analytical method to the environment and human health.

#### Practicability metric approach (BAGI)

The analytical method's performance is assessed by the BAGI tool [32]. This tool considers ten attributes related to the technique, the instrument, and the samples. A score and a pictogram are produced. In our method, several samples can be simultaneously analyzed within an hour; a simple extraction procedure, a semi-automated instrument, and commercially available solvents were used. The size of each sample was 5 mL, containing 1 mL of plasma. As displayed in Table 6, a pictogram and a score of 75 were produced with a light blue shade, which indicates the greenness of the developed TLC method and that it is an eco-friendly analytical method for the environment and human health.

#### Whiteness assessment

The Whiteness assessment of a method was done by assessing three aspects [41–45], including execution, greenness, and workability. In the red–green–blue (RGB) Excel sheet downloaded from the original article describing the assessment method [33], several parameters are given scores according to the degree of compliance with the required values. The method achieved the scope of the application, had low LOD and LOQ values, and had acceptable accuracy and precision, so it got a score of 100 for the execution aspect. The total number of pictograms for the used solvents was 9, which wasn't too high; the waste amount was over 100 mL that wasn't treated; the energy consumption was moderate, and the occupational hazards were low, so the greenness score was 90. Finally, the method was cost-, time-, and energy-efficient; the procedure was simple, although it could not be miniaturized. A score of 95 was given for workability. The overall score of whiteness was 95, as found in Table 6**.**

### A brief comparison between the developed and the reported TLC methods

The developed TLC method was compared briefly with the previously published chromatographic methods for determining PRP and MTZ in plasma matrices. As illustrated in Tables 7, 8 the proposed method has advantages over the previously published methods of determining PRP and MTZ in spiked human plasma. It is more accurate than the reported methods [11–13, 21, 25, 26] for estimating PRP and MTZ. In addition, the proposed method is more precise than the reported ones [11, 12, 26] for determining the two drugs. Moreover, the proposed method used greener mobile phase solvents than the reported methods [11, 13, 21, 25, 26], which used chloroform and acetonitrile (toxic and non-green), proving that the developed method is greener and safer for the environment and human health. On the other hand, the proposed method has the drawback of being less sensitive than the reported methods [11–13, 21, 25, 26] for the determination of PRP and MTZ.

**Table 7:** A brief comparison of the regression parameters and chromatographic conditions between the developed TLC method and the reported LC methods for the determination of propranolol and/or methimazole

The Method	The matrix	Calibration range (µg/band)	LLOQ (µg/band)	Accuracy and precision	Mobile phase	Detection wavelength	R_f_/t_R_ of PRP or/and MTZ
1. The proposed TLC method	MTZ and PRP in spiked human plasma using HCA as IS	0.1–1.2 (MTZ) 0.06–1.2 (PRP)	0.10 (MTZ) 0.06 (PRP)	Accuracy: between (96–100) % for MTZ, and (100.4–104.5) % for PRP Precision: between (1.8–3) % for MTZ, and (1–4) % for PRP	Ethyl acetate: acetone: 33% NH_3_ solution (9: 1: 0.05, by volume)	UV detection at 254 nm	MTZ = 0.62 PRP = 0.19
2. Reported HPLC method [11]	MTZ in plasma	0.005–0.025 (MTZ)	0.005 (MTZ)	Accuracy: between (93–107) % Precision: between (2.7–7.5) %	Chloroform	UV detection at 405 nm	MTZ = 5 min
3. Reported LC–MS/MS method [12]	MTZ in human serum or plasma	0.001–0.1 (MTZ)	0.001 (MTZ)	Accuracy: between (89.5–101.1) %, Precision: between (1.4–10.2) %	Gradient elution of (A) 10 mM NH_4_-acetate, and (B) methanol	Tandem mass spectrometry (MS/MS)	MTZ = 5.53 min
4. Reported GC method [13]	MTZ in plasma	0.015–0.15 (MTZ)	0.015 (MTZ)	–	Nitrogen gas	Mass spectrometry (MS)	MTZ = 4.2 min
5. Reported TLC method [21]	PRP in human serum using verapamil as IS	0.005–0.1 (PRP)	0.005 (PRP)	Accuracy: between (96.35–98.82) %, Precision: between (1.84–2.85) %	Chloroform: methanol: ammonia (9: 1: 0.04, by volume)	UV detection at 290 nm	–
6. Reported HPLC method [25]	PRP in human plasma using diltiazem HCl as IS	0.02–0.28 (PRP)	0.02 (PRP)	Accuracy: between (79–197) % Precision: between (1- 1.5) %	Acetonitrile: pH 4.5 phosphate buffer (35: 65, v/v)	UV detection at 214 nm	PRP = 6.6 min
7. Reported HPLC method [26]	PRP in human plasma	0.015–0.18 (PRP)	0.015 (PRP)	Accuracy: between (97.9–102.8) % Precision: between (1.5–5.7) %	160 ml water, 180 ml methanol, 70 ml acetonitrile, 2.5 ml acetic acid, and 125 µl triethylamine (by volume) at pH 3.4	UV detection at 291 nm	PRP = 9.1 min

**Table 8:** A comparison between the developed TLC method and the reported chromatographic methods for the determination of propranolol and/or regarding greenness and applicability

The Method	GAPI	AGREE	BAGI
1. The proposed TLC method			
2. Reported HPLC method [11]			
3. Reported TLC method [21]			
4. Reported HPLC method [25]			

## Conclusion

Using HCA as an internal standard, a sensitive green TLC technique was created to simultaneously measure PRP and MTZ in their combination and spiked human plasma. For MTZ, being toxic upon long-term usage and being synergized with PRP created an urgent demand for developing such an assay. The process has the benefits of being quick, easy, affordable, and environmentally friendly, and it requires a small number of samples and reagents. All validation parameters met the US-FDA acceptance criteria for verifying bio-analytical methods. The newly suggested TLC approach is environmentally friendly. It has little to no negative impacts on human or natural life, according to a greenness evaluation conducted using three different tools.

## Data Availability

The datasets used and/or analyzed during the current study are available from the corresponding author on reasonable request.
